# Automatic Fruit Morphology Phenome and Genetic Analysis: An Application in the Octoploid Strawberry

**DOI:** 10.34133/2021/9812910

**Published:** 2021-05-12

**Authors:** Laura M. Zingaretti, Amparo Monfort, Miguel Pérez-Enciso

**Affiliations:** ^1^Centre for Research in Agricultural Genomics (CRAG), CSIC-IRTA-UAB-UB, 08193 Bellaterra, Barcelona, Spain; ^2^Institut de Recerca i Tecnologia Agroalimentàries (IRTA), 08193 Barcelona, Spain; ^3^ICREA, Passeig de Lluís Companys 23, 08010 Barcelona, Spain

## Abstract

Automatizing phenotype measurement will decisively contribute to increase plant breeding efficiency. Among phenotypes, morphological traits are relevant in many fruit breeding programs, as appearance influences consumer preference. Often, these traits are manually or semiautomatically obtained. Yet, fruit morphology evaluation can be enhanced using fully automatized procedures and digital images provide a cost-effective opportunity for this purpose. Here, we present an automatized pipeline for comprehensive phenomic and genetic analysis of morphology traits extracted from internal and external strawberry (*Fragaria x ananassa*) images. The pipeline segments, classifies, and labels the images and extracts conformation features, including linear (area, perimeter, height, width, circularity, shape descriptor, ratio between height and width) and multivariate (Fourier elliptical components and Generalized Procrustes) statistics. Internal color patterns are obtained using an autoencoder to smooth out the image. In addition, we develop a variational autoencoder to automatically detect the most likely number of underlying shapes. Bayesian modeling is employed to estimate both additive and dominance effects for all traits. As expected, conformational traits are clearly heritable. Interestingly, dominance variance is higher than the additive component for most of the traits. Overall, we show that fruit shape and color can be quickly and automatically evaluated and are moderately heritable. Although we study strawberry images, the algorithm can be applied to other fruits, as shown in the GitHub repository.

## 1. Introduction

Demographic pressure and climate change are two of the major challenges of the 21st century. The worldwide population continues growing exponentially, and it is expected to reach ~9.8 × 10^9^ in 2050 [1]. Climate change generated by greenhouse gas emissions is possibly the greatest threat, as it is leading to extreme weather conditions, increasing areas of drought, and species extinction, among others [2–4]. In this adverse context, food production needs to be increased significantly. Increasing food production is not enough though. Breeding programs should also consider food safety and environmental care among their objectives [5, 6].

Artificial breeding is mainly responsible for the dramatic rise in food production we have witnessed for over a century. The main goal of plant and animal breeding is to utilize genetic variability of complex traits to increase performance and optimize use of resources. A current bottleneck in plant breeding programs is the evaluation of hundreds of lines under different environmental conditions [7, 8]. Plant breeding involves both genomics and phenomics, i.e., the expression of a genome in given environments. While available technologies can routinely and inexpensively scan the genome, high-throughput phenotypic characterization remains a difficult task [9, 10]. Automatizing phenotype measurement is then needed to increase the pace of artificial selection and is, unsurprisingly, one of the main targets of “Precision Agriculture” [11, 12].

The term “phenomics” or “phenometrics” was coined by Schork [13] as an attempt to understand events happening in between full genome and clinical endpoint phenotypes in complex human diseases. The expression quickly spreads to animal and plant breeding research as a concept that bridges the gap between genotypes and the “end-phenotypes.” Although the term phenomics was devised in line with “genomics,” that is, to describe the whole phenome of any organism, note that the phenome varies over time and between cells or tissues and can never be fully portrayed [14].

Although electronics applied to agriculture has a long history, a window of opportunities has emerged in the phenomics field with recent improvements in robotics, electronics, and computer science. The subjective, time-consuming, and often destructive human data collection is being replaced by miniaturized, cheap sensors, digital cameras, cell phones, unmanned aerial vehicles, and mass spectrometry, among others, that allow collecting hundreds of phenotype data objectively and inexpensively [9, 15–17]. The challenge now is to develop new and improved analytical tools, capable of transforming this wealth of data into valuable knowledge [15]. This is a rapidly evolving field, and numerous software and pipelines to automatize phenotype collection are already available [18–22]. Many of these tools focus on the analysis of root images and, as far as we know, require more user intervention than we propose, making it impractical to analyze hundreds of images.

Digital images are among the cheapest and most widely available types of data. Imaging allows assessing morphological traits, which are highly relevant in numerous plant breeding schemes, since they can critically affect consumer acceptance especially in fruits [23–25]. Nevertheless, consumer preferences on appearance traits differ around the world and between communities. Like most traits, fruit shape is determined by genetic and environmental factors such as flower morphology or insect-mediated pollination [26, 27]. In all, morphological traits are among those with the highest heritability, which has allowed breeders to rapidly modify shape, size, and color patterns of agricultural products [20, 28–30].

Although numerous works have been developed in the area of fruit morphology, most of them have focused in the inheritance of linear measures, e.g., diameter, perimeter, and circularity [20, 31–33]. By definition, however, morphological traits are highly dimensional. Computing only linear, univariate phenotype leads to a loss of information by extremely simplifying the features of a shape [28, 34]. The use of geometric-morphometric approaches for shape analysis is warranted [35]. Further, fruit shape has been traditionally evaluated subjectively [36] but can be enhanced by resorting to automatized procedures. For instance, hundreds of fruit pictures can be routinely and inexpensively collected, even in the field, with a cell phone camera. Automatized image processing and analysis can then dramatically change the way shape and color traits are collected and characterized.

Here, we present a comprehensive phenomic and genetic analysis pipeline for fruit morphology automatic analysis. Two main issues are addressed: (1) converting the raw data (fruit images) into a processed curated database and (2) designing an efficient analysis workflow to analyze the fruit shape and color phenome. Finally, genetic parameters are automatically inferred from pedigree information. We apply the pipeline to images of cultivated strawberry (*Fragaria x ananassa*) fruits. In addition to previous similar works in strawberry, e.g., Feldmann et al. [18], we provide a wholly automatized pipeline and new tools to analyze shape and color patterns.

## 2. Materials and Methods

### 2.1. Plant Material and Imaging Acquisition

Lines employed are part of the strawberry breeding program of the Planasa company (https://planasa.com/en/) and are routinely used to develop new elite genotypes. The experiment consisted of 24 crosses between 30 parental lines of *F. x ananassa*. We evaluated 20 randomly chosen lines per cross for all but 2 crosses, for which we chose 19 lines at random. A total of 478 seedlings and 30 parental genotypes were evaluated (Supp Table 1). Shape varied between the cultivars studied, e.g., circular, ellipsoid, or rhomboid, and color ranged from white to dark red.

Strawberries were grown in plastic semitunnel using standard cultivation practices in South West Spain (Huelva, 37°16′59^″^N, 7°9′18^″^W). Fruits were collected from two individual plants of each line at the end of April 2018 in only one harvest event. Fruits from both plants were pooled in the photographs. We took images of 1 to 7 sliced fruits per genotype using a Nikon D80 digital camera. Samples were laid on a black surface, with the camera positioned at 35 cm height. The focal length was 18 mm, the manual aperture was f/8, and the exposure time was 1/8 seconds. Illumination consisted of two white light sources at both sides of the camera. In total, we took 508 images of 3872 × 2592 pixels that contained all external and internal sides of fruits and the label for each genotype in the same image.

### 2.2. Preprocessing and Segmentation

**Figure 1:**
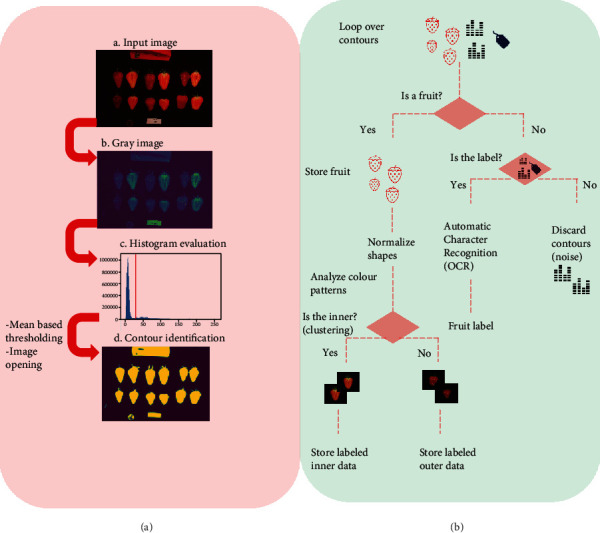
Workflow for automatic segmentation and label recognition from strawberry images: (a) feature extraction; (b) feature preprocessing and database generation.

The first step in the pipeline is to segment and recognize the objects, since each raw image contains internal and external fruits, a rule, a coin, and a printed genotype (the strawberry line) label. Image segmentation is needed for obtaining meaningful morphometric and color information. However, most of available technologies to determine the boundaries of the objects at the pixel level are usually semiautomatic and time-consuming [37–40]. Our fully automatic python-based pipeline takes the images of each strawberry line and outputs a curated database of square images (1000 px) and reads the genotype label (Figure 1). https://github.com/lauzingaretti/DeepAFS/blob/main/main.ipynb explains how to apply the most expensive part of this workflow to alternative experiments. Note that after creating a curated database, a standard multivariate analysis can be easily run using R/Python tools to shape evaluation.

For segmentation, the three-channel digital signals (RGB/BGR) are converted into grayscale and blurred using Gaussian filtering of size 5, to remove undesirable noise. The histogram information is used for image binarization, i.e., splitting the background and foreground. Here, we binarized the image using simply the mean value of the pixel as a threshold. The pipeline also allows Otsu thresholding [41], which is designed to automatically define the threshold by minimizing the “overlap” between two classes. After binarization, we performed erosion and dilation, the former shrinks the edges, and the latter makes the image region grow. Finally, the algorithm extracts the regions of interests (ROI) and determines whether it is a strawberry or an image label. The color pattern analysis allows us to classify the internal or external part of a fruit image. We here apply a *k*-means clustering based on the information about the color mean, color standard deviation, and the ratio between them for all the fruits; i.e., we compute these 3 features for all the fruits, and then, we classify these observations into 2 clusters to split the internal and external part of the fruits. For the labels, the Optical Character Recognition (OCR) algorithm from PyTesseract library (https://pypi.org/project/pytesseract/) is used to read the genotype name and automatically label the image into the database. As a result, the algorithm delivers a curated database of 508 folders labeled with the name of each genotype and subfolders containing either the internal or external strawberry pictures (Figure 1, Algorithm 1 in Suppl. Info). All fruits are stored in square images (1000 px size or user-defined), with the fruits placed in the center and filled with black pixels.

### 2.3. Automatic Fruit Phenotyping

**Figure 2:**
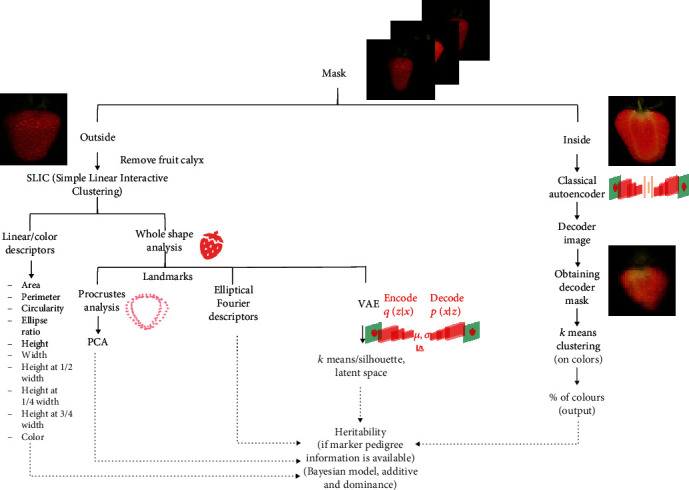
Data analysis workflow. The input is the segmented internal and external fruit images from workflow in Figure 1. External images are used for linear and multidimensional shape analysis through different standard and machine learning approaches, including deep learning. Internal images are used to estimate the color pattern of the internal fruit section. Additive and dominance genetic components of each of the extracted morphometric and color phenotypes are estimated using Bayesian Linear Modeling using either pedigree or DNA marker information. Code available at https://github.com/lauzingaretti/DeepAFS

Once masks for either internal or external fruit images are obtained, an automatic phenotyping procedure is run for inside or outside parts separately (Figure 2). Classical linear descriptors and multivariate and deep learning techniques are combined from a novel perspective to dissect a variety of shape and color patterns. If pedigree or marker information is available, a genetic analysis can be employed to estimate variance components for each of the fruit phenotypes. In the following, we describe the main methods implemented in the pipeline of Figure 2.

### 2.4. Autoencoder and *k*-Means to Infer Internal Color Patterns

**Figure 3:**
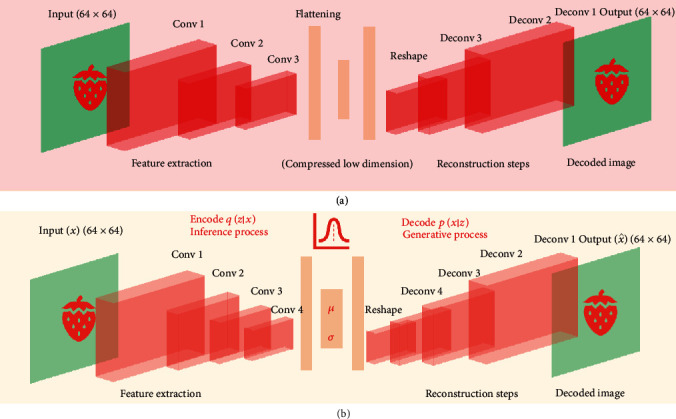
Autoencoder architectures: (a) architecture of convolutional autoencoder applied to the internal fruit images; (b) architecture of convolutional variational autoencoder applied to external fruit. Unlike classical autoencoders, variational autoencoders are generative process as they learn the parameters of a distribution, instead of the feature representation. The last network was trained using an image of size 64 × 64, the encoder step consisted on 4 convolutional layers with a kernel size equal to 3, and the linear rectified “ReLU” as activation function to perform feature extraction (see details in GitHub account). Finally, the convolution output is flattened, and the mean and sigma parameters are extracted from a dense layer. In the last network, the decoder step starts with a vector sampled from the latent distribution as input and reconstructs the input by performing deconvolution operations. The last deconvolution uses the sigmoid as activation function. The loss function is the Kullback-Leibler (KL) divergence, which consists of both a “reconstruction” and a “regularization” term. The first network is a classical autoencoder, which uses the classical mean squared error (MSE) as loss function.

We used an “autoencoder” (AE) network to perform an unsupervised clustering of the internal images. An autoencoder (Figure 3(a)) is an unsupervised machine learning technique that applies backpropagation to train a neural network where the outputs are the same values as the inputs [42]. The AE gives new insight into image analysis by learning the structure about the data; i.e., it is not designed to copy an exact replicate of the input but instead to learn the repeatable and most useful properties.

We used a convolutional AE, as convolutional operations are especially suited for image analysis [42, 43]. These layers create a feature map from the input image, preserving the relationships between pixels in the original space (Figure 3(a)). Each convolution outputs a scored-filtered image, where a high score means a perfect match between the original image and filtered image. The output layer is obtained by applying the Rectified Linear Unit (ReLU) activation function. Finally, as usual, in any convolutional architecture, a max-pooling layer shrinks the output size and achieves a smoother representation, summarizing adjacent neuron outputs by computing their maximum (see accompanying GitHub).

The decoded images from an AE architecture are less noisy than the original ones, making it easier to detect repeatable/consistent color patterns. Our approach consists in taking five colors as reference: a class for the background (black) and four classes for the internal fruit color patterns, including calyx. The four “reference classes” were “orange-like” (198, 99, 35, in RGB coordinates), “quasired” (184, 46, 8), “pale” (194, 144, 78), and “green” (76, 75, 20) for sepals. We then perform a *k*-means clustering with *k* = 4 after removing the background, and we assigned each cluster to the nearest reference color using the Euclidean distance between the average color of each cluster in the sample and the reference coordinates. As a result of this step, the surface of each of 1900 strawberry images is split into four categories of colors.

### 2.5. Superpixel Algorithm to Remove the Calyx

Some of the fruit pictures contain sepals that interfere with fruit shape quantification and need to be removed prior to estimating shape parameters. For that purpose, we applied the Simple Linear Interactive Clustering (SLIC) algorithm [44] from the Python skimage library. SLIC is based on the “superpixel” concept. Basically, a superpixel is a group of pixels sharing perceptual and semantic information; e.g., the pixels in a superpixel are grouped together because of their color or texture features. The iterative algorithm starts with regularly spaced *K*-centers at a given distance, user defined as *S*, which are then relocated in the direction of the lowest gradient in a 3 × 3 neighborhood window to avoid being at the edges of the image. Further, a pixel is assigned to a given cluster if its distance to the cluster's center is smaller than the distance to the other centers in the search area, as determined by *S*. Finally, the centers are recalculated by averaging all the pixels belonging to the superpixel. The iterative process ends when the residual error (distance between previous centers and recomputed ones) does not exceed a fixed threshold. SLIC outputs a set of meaningful clusters, splitting the background, the calyx, and the fruit. Knowing that all our fruits are centered in the image (the segmentation procedure outlined in Figure 1 ensured that every image was centered), the superpixel containing the central pixel matches with the fruit.

### 2.6. Univariate Phenotypes: Linear Descriptors

Numerous object shape descriptors exist in the literature. Particularly for fruits, a controlled vocabulary was established in Brewer et al. [20]. Here, we implement a custom script to compute some standard linear measures: circularity, solidity, shape aspect [32], ellipse ratio [20], fruit perimeter and area, fruit width at 25% height, fruit width at 75% height, fruit width at 50% of height, total height, and maximum width. Circularity is a measure of the degree of roundness of a given object, defined as the ratio between the area of a given object and the area of a circle with the same convex perimeter; i.e., a value near one means a “globe” o “circular” shape. Solidity is the ratio between the area of the object and the area of the convex hull of a given shape. Most of the linear descriptors used here are standard in fruit shape analyses [18, 20, 32, 39, 45].

The external fruit color was measured using the CIELAB space, where *L* indicates the luminosity and *a* and *b* are the chromatic coordinates. The variation on the index *a* indicates the transition between green to red, where a higher value means a redder object. Variations in *b* reflect the change between yellow and blue colors, i.e., a higher *b* value refers to a “bluer” object.

### 2.7. Generalized Procrustes Analysis (GPA)

**Figure 4:**
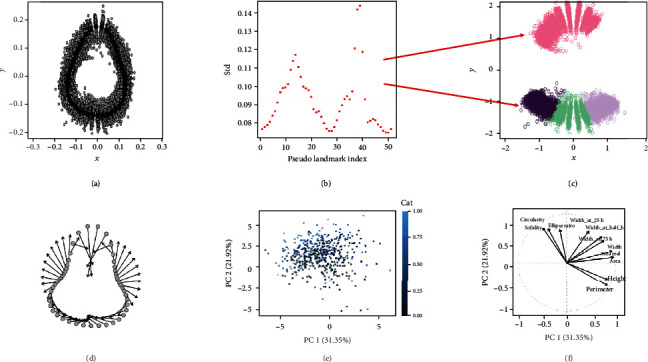
Summary of main analyses performed. (a) Generalized Procrustes Analysis output: landmarking superimposition for all external fruit shapes. (b) Standard deviation of each of the 50 landmarks; the dotted line parallel to the *x*-axis corresponds to the average standard regression coefficient. Landmarks with a coefficient above the average are the most variable regions. (c) The most variable regions, which determine the fruit shape, are the tip, the neck, and both sides around the neck. (d) Two extreme Procrustes analysis plots: minimum and maximum consensus for the fruit shape. (e) PCA of all linear shape descriptors; each dot represents a different sample, and the color is proportional to the predicted proportion of fruit to each category from the clusters obtained by variational autoencoder. (f) Relationship between the linear shape variables from the PCA.

Shape is usually defined as all the geometric information that remains unchanged after filtering out the location, scale, and rotation effects of a given object [28]. The above shape linear descriptors are standard in the literature but do not provide a whole shape portrayal. Alternatively to linear descriptors, shape variations can be described using “pseudolandmarks” [35], which identify points around the outline of the object. Here, 50 pseudolandmarks were defined as the intersection between 50 equally spaced conceptual lines starting from the centroid and the fruit contour (Figure 4(a)). Next, we performed a Procrustes analysis [46]. The Procrustes analysis is aimed at finding the transformation T such that given two matrices *X*_1_ and  *X*_2_, the product *X*_2_*T* best matches *X*_1_. The Generalized Procrustes Analysis (GPA) is an extension of the method devised to align many matrices simultaneously [46]. In a morphometric analysis, this is done by averaging the distance between all the landmarks on a target shape and the corresponding points on a reference. The pseudolandmarks of the samples can then be analyzed as a multivariate object using, for instance, a principal component analysis (PCA). In addition, the pseudolandmark variability gives insight on the most important regions that determine the differences between shapes. We used the Momocs [47] and geomorph [48] R packages to run these analyses.

### 2.8. Elliptical Fourier Descriptors

An alternative approach to morphometric analysis is elliptical Fourier transformation [49]. This method describes a closed curve as a sum of sine and cosine functions of growing frequencies. As its name suggests, Fourier harmonics are ellipses, and a larger number of harmonic means that more ellipses are fitted to a given contour. The second-order harmonic is simply one ellipse with the values of sine and cosine components for the *x*- and *y*-axis, respectively. As the strawberry fruit is a relatively simple shape, four harmonics were enough to describe all the shapes in the database, giving a total of 16 coefficients. A PCA of the Fourier components can also be employed to quantify morphometric variability, as in the Procrustes analysis. Geomorph [48] R package was employed for this purpose.

### 2.9. Conditional Variational Autoencoders (VAE) to Cluster Shapes

Fruit shape can also be addressed from a completely different angle, such as obtaining clusters of shapes to objectively classify fruits in groups of similar morphology [18]. A standard approach consists of flattening the image and grouping the raw data, treating each pixel as a feature. Unfortunately, clustering algorithms are not exempt from the “curse of the dimensionality” problem [50] and they perform poorly as the number of analyzed dimensions increases, especially if noise is high.

A natural way to solve the aforementioned issue is to apply a dimension reduction technique before clustering. Although the classical autoencoders seem to be a good option, as shown above, AEs were conceived to perform a nonlinear and not isometric dimensionality reduction, and thus, they do not preserve the geometrical properties of the original space [51]. Unlike traditional autoencoders, variational autoencoders (VAEs) [52–54] preserve distances and, importantly, are generative models (Figure 3(b)). The main difference between AE and VAE is that the latter encodes the input as a distribution over a latent space. Basically, given an input *x*, VAE creates a latent distribution *p*(*z* | *x*) and the input reconstruction *d*(*z*) is obtained after sampling *z* from the latent representation *z* ~ *p*(*z* | *x*). The VAE does not only force the latent space to be continuous; it can also generate meaningful information, even with images that it has never seen before.

The key aspect in VAE training lies in the loss function, which includes a “reconstruction” and a “regularization” term. The former is the usual loss or the joint log-likelihood between the true and the VAE output, whereas the second is the entropy corresponding to the Kullback-Leibler divergence [42] between the latent distribution *N*(*μ*_*x*_, *σ*_*x*_) and the standard normal distribution *N*(0, 1). Without incorporating a regularization, the VAE behaves as AE, where the latent space is neither complete nor continuous. Regularization forces the latent distribution to be close to the normal standard, generating a continuous space of low variance centered in the origin, which is suitable for data clustering and generation [42].

Here, we run standard *k*-means clustering of the latent space, with *k* varying between 2 and 9 groups. We chose a maximum *k* = 9 given that up to nine strawberry shapes have been proposed in the literature, in particular in the Japanese market [55]. We assessed the cluster robustness using the silhouette index [56]. This index determines how well each object fits into its cluster, taking into account intra- and between-class variations. The index ranges between -1 and 1, and a value close to 1 means that the cluster is compact and homogeneous. Importantly, the combination of VAE and clustering also allows us to use conditional VAE to generate the expected fruit pertaining to a specific group.

### 2.10. Genetic Parameter Inference

Genetic parameters determine how successful artificial selection will be and are therefore a critical parameter of any plant breeding scheme. Heritability (*h*^2^) is the proportion of phenotypic variance explained by the genetic variation [57]. To estimate *h*^2^, the degree of resemblance between relatives using the pedigree was used (see Supp Table 2). Take linear model

(1) $$\begin{array}{c} y=\mu +a+d+\varepsilon , \end{array}$$

where **y** represents the phenotype vector, averaged for each genotype; *μ* is the intercept; **a** ~ *N*(0, **Aσ**_*a*_^2^), **d** ~ *N*(0, **Dσ**_*d*_^2^), and **e** ~ *N*(0, **Eσ**_*e*_^2^) are the additive effects and dominance effects; and **ε** ~ *N*(0, **I***σ*_*ε*_^2^) is the residual component, respectively; **A** = {*a*_*ij*_} and **D** = {*d*_*ij*_} are the additive and dominance covariance matrices, respectively. Both **A** and **D**can be computed recursively from the pedigree [58]. In the presence of marker information, **A** and **D** can be computed as specified in [59, 60] and implemented in [61] but statistical inference is otherwise identical. Posterior distributions of the genetic parameters were obtained using Reproducing Kernel Hilbert Space (RKHS) regression with the BGLR package [62]. The additive and dominance variance fractions were estimated as ${\hat{h}}_{a}^{2}=s_{a}^{2}/\left(s_{a}^{2}+s_{d}^{2}+s_{\varepsilon }^{2}\right)\text{ and }{\hat{\;h}}_{d}^{2}=s_{d}^{2}/\left(s_{a}^{2}+s_{d}^{2}+s_{\varepsilon }^{2}\right)$, where *s*_*i*_^2^ is the mean posterior estimate of *σ*_*i*_^2^.

## 3. Results

### 3.1. Shape Descriptors

Shape linear descriptors, pseudolandmarks, and elliptical Fourier transforms for fruit shape were computed for the 1920 external images output from pipeline in Figure 1 and Algorithm S1.  Figure 4(d) shows the minimum and maximum consensus for shape superimposition, suggesting that shapes vary between a “globose-like” to an “elongated-like” form in these samples. The standard deviation of the first PCA from GPA coordinates (Supp Fig. 1) of the tip, neck, and both sides around the neck is above the mean (Figure 4(c)). This suggests that these regions are responsible for the main shape variations in strawberry, in agreement with Feldmann et al. [18]. Supp Fig. 1 shows the fruit shape variations from the Procrustes principal component analysis (Proc-PCA). The first principal component describes the variations between “elongated”- to “globose”-like. Observations with a negative score on that component correspond to elongated fruits, while those who have positive scores are “globose”-like fruits. A permutation-based Procrustes analysis of variance was conducted to assess the effect of the crosses on the fruit shape. The *p* value obtained after 100 permutations shows a significant effect of the lines, i.e., genotypes, in the fruit shape (*p* < 0.01), suggesting that the shape is heritable (Supp Table 3).

We also set a fourth-order elliptical Fourier to describe the main strawberry shape variations (see Supp Figs. 2 and 3). As in the Procrustes analysis, variations in the first principal component of the elliptical analysis show that the strawberry shapes vary between “globose-like” to “elongated-like” (see a few examples in Supp Fig. 7). Similarly, the first component from elliptical PCA can also be used as a “morphological” descriptor. A *k*-means clustering using the two first PCA components of Fourier transform similarly detects the two previously defined groups of shapes when setting *k* = 2 (Supp Fig. 4).

**Figure 5:**
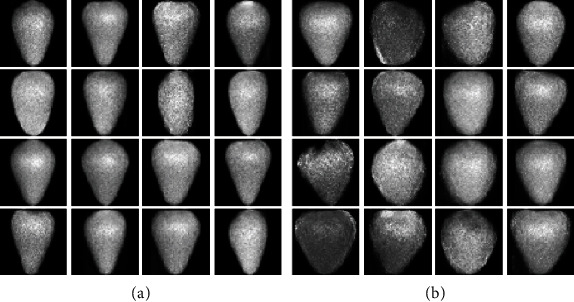
Images generated using the variational autoencoder combined with *k*-means in the latent space with *k* = 2: (a) images from the “elongated-like” cluster; (b) images from the “globose-like” cluster.

Alternatively, one can directly identify the number of different shapes from a collection of images. We used a VAE (Figure 3(b)) to automatically discover the optimal number of shapes in our database, which again was *k* = 2 (Supp Fig. 5 and 6). About 35% of the strawberries belong to the “globose-like” shape, whereas the remaining fruits were classified as “elongated-like” (Figures 5(a) and 5(b)).

Figure 4(e) shows a PCA on the linear descriptors, where the color of each sample is proportional to the predicted cluster probability. A dark color corresponds to a fully elongated shape, and a light blue, to a fully round fruit. Note that shape gradient is mainly observed along the second principal component. Interestingly, the most influential variables in this component are the fruit ratio between main and minor ellipse axis, the circularity, and solidity coefficients (Figure 4(f)). All of these are shape-related variables. It is not surprising that solidity and circularity are highly correlated, since the convex hull area increases when a shape digresses from a circle (circularity), and solidity approaches zero. The area, perimeter, and height are quasi-independent of the aforementioned descriptors and are not related with the shape clusters.

### 3.2. Color Descriptors

For the external side color in our dataset, the *L* channel ranged between 7.01 and 118.30, mean of 75.54; the *b* channel ranged between 127.9 and 184.8, mean value of 167.1; and the *a* channel had a mean of 175.4, ranging between 128.8 and 192.6.

**Figure 6:**
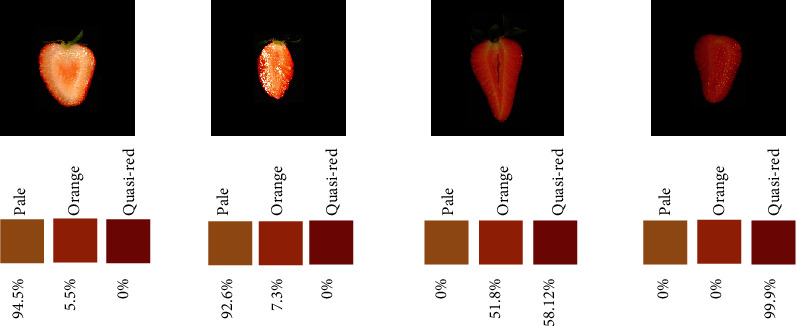
Estimated percentage of each of the three reference colors in four picked strawberries.

Estimating the color of the internal fruit is more challenging than that of external parts, as it fluctuates in a wider range of patterns.  Figure 6 shows the estimated percentages of each reference color for four chosen strawberries. Note that the percentage of “quasired” is zero and most of the fruit is computed as “pale” (~95%) for the first two, whitish fruits. Two colors, “quasired” and “orange-like,” predominate in the third fruit. Finally, the last fruit is almost red, as can be verified from the estimated quasired value (99%).

### 3.3. Genetic Parameter Estimation

**Figure 7:**
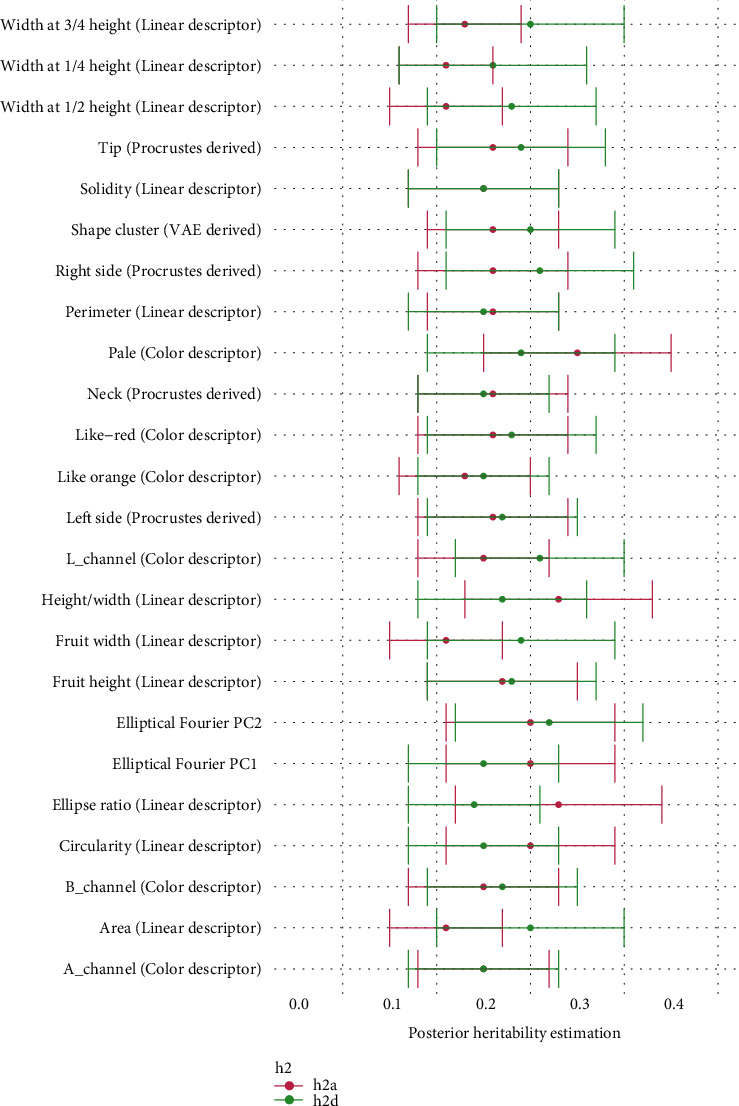
Estimation of additive (h2a, in pink) and dominance (h2d, in green) variance fractions for measured traits. Additive and dominance effects were calculated as indicated in Genetic Parameter Estimation.

Figure 7 shows the Bayesian estimates of heritability for all automatically extracted traits. We used the pedigree information to compute the additive and dominance relationship matrices, since we did not have genotypes. Like many polyploid species, strawberry is clonally propagated [63]. Inferring the dominance component in these cases is critical, as clonal propagation allows a straightforward utilization of gene interaction [64]. Interestingly, we found that dominance variance was higher than the additive component for most of the traits. The sum of both components ${\hat{h}}_{a}^{2}+{\hat{h}}_{d}^{2}$ ranged between 0.4 and 0.6, indicating that the traits are clearly heritable. The ellipse ratio and the ratio between height and width were the most heritable characters, exhibiting an important additive component. Elliptical Fourier components, as well the percentage of fruits of each of both categories obtained from VAE, also have a high heritability, for both additive and dominance components. Regarding the internal color, we find that the pale color has an important dominance component.

## 4. Discussion

Over the last decades, plant and animal breeding programs have benefited from the development and cost reduction on genomic technologies [65, 66]. Breeding nevertheless depends of both genotype and phenotype, and our ability of characterizing the latter is much more limited compared to the former [9, 10]. In fact, one of the biggest challenges of “Precision Agriculture” is to transform large-scale datasets collected with sensors into phenotypic measurements that can be used for genetic improvement.

Consumer attitudes are increasingly shaping agricultural practices. In the case of fruits, consumer preferences are based primarily on fruit appearance. However, measuring this trait is not straightforward, as it is a complex mixture of shape and color patterns. A crucial aspect for improving appearance is then to characterize the color and shape of the fruits in an inexpensive and fast way. In this paper, we deliver a fully automatized pipeline that analyzes fruit appearance as complex multivariate data. While this is not the first study characterizing fruit shape variations, our procedure is quite more automatized than their predecessors as it requires minimal human intervention [18, 20, 40]. It also incorporates new features such as the use of variational autoencoders (VAE) to automatically detect the most likely number of underlying shapes or to cluster the internal color.

The pipeline presented here or previous efforts to automatize fruit morphology measurement by Feldmann et al. [18] are important steps to increase agriculture efficiency. They are by no means sufficient, and additional developments are warranted. A first limitation is that algorithms need to be trained in the specific dataset that will be used in production and can sometimes be difficult to generalize to different scenarios. A second limitation concerns the phenotypes measured. For instance, uniformity of shape and lack of blemishes (like depressions or creases) significantly impact the value of the product but were not studied here. Uniformity of fruits can be easily quantified, e.g., measuring the dispersion along landmarks (Figure 4(a)), whereas irregularities in color that may mean fruit damage can be more challenging. In the lab, as done here, perhaps, a suitable color clustering to associate color patterns with fruit damages could be envisaged. To be really useful, however, fruit damages should be evaluated once the product has been packaged, prior or after distribution, which would need distinct code from that employed here. The number of seeds is also important economically, but we found that a very high resolution is needed to quantify them. Finally, 3D approaches have also been evaluated in fruits, including strawberry [67, 68]. Three-D imaging is far more demanding in terms of sample collection and computationally than 2D [69, 70]. This hampers using 3D technologies as massively as 2D, although 3D has a number of advantages, mainly a far more realistic and comprehensive fruit representation. For instance, Li et al. [71] utilize 3D imaging to assess fruit uniformity and show that it can be characterized by combining up to six linear parameters.

Our algorithm requires images being taken on a homogeneous black or white surface, and field images are not allowed. To compare the shapes and colors, all shots must be taken in the same conditions, using the same digital camera placed at the same height and setting the same parameters: focal length, manual aperture, exposure time, and lighting. Scanned images are also allowed but the same scanning conditions must be followed in all images.

Although 2D digital images are among the easiest phenotypes to collect, analyzing them can be challenging, partly because object boundaries must be determined, a process known as feature extraction. Numerous classical [41, 44, 72] and deep learning approaches [73, 74] have been developed in computer vision and image processing to meet this objective. Here, we combined some of these methods to automatically segment fruit snapshots and read the fruit label. The main approach we used is not new, as it is based on an algorithm developed in the late seventies [41]. However, we resort to novel techniques in order to remove undesirable image noise [75], and we characterize color pattern or classify fruits through a variational autoencoder (Figure 3) [42].

In this work, we characterize shape and color variations using several complementary methods, from naïve linear descriptors to multivariate and deep learning techniques. It is important to point out that results from all approaches are consistent and suggest that the fruits in our database can be classified into two groups, “globose-like” and “elongated-like” (Supp Figs. 5 and 6). We determine that the most variable regions are the neck, neck sides, and the tip of the fruit (Figures 4(b) and 4(c)). The “shape” linear descriptor, i.e., the ratio between fruit shape and height, is a good morphological descriptor (Figures 4(e) and 4(f)) and is as discriminative as more complex multivariate characterizations. An ANOVA on the Procrustes coordinates shows that genotype is significant (*p* value < 0.01, Supp Tab 2), another indirect indication that shape is heritable.

Shapes can be classified using standard clustering techniques with the number of clusters *k* previously specified, as shown by Feldmann et al. in strawberry [18]. Our results are in agreement with these authors' in that we also find that shape is heritable and that a few components may be needed to classify shapes (Figure 4(f)). In addition to that approach, here, we propose a completely unsupervised manner based on variational autoencoders (Figure 3). The advantage of this analysis is that shape discovery not only can be automatized but also is capable of generating shapes not seen before. Predicting shapes and appearance of new genotypes can be a powerful tool to design new crosses, as the breeder can evaluate not only the average shape but also their variability in morphology. To our knowledge, VAEs have not been utilized for these purposes yet.

Here, we have explored multiple methods to describe fruit morphology. Although somewhat redundant, each metric has its own advantages and limitations. For instance, previous works (e.g., [18, 20]) show that the classical linear descriptor defined as the ratio between height and width is an accurate way to describe fruit shape variations in fruits like strawberries. The advantage of using this method lies in the principle of parsimony; i.e., it is the simplest way to characterize a shape. Albeit its simplicity, this measure is not complete enough to describe the many variations that can occur, since many vegetables and crops, like tomatoes, melons, cucurbits, and even strawberries, exhibit enormous morphological variations [30]. In these scenarios, multivariate descriptors (like Fourier and Generalized Procrustes) are more suitable for the analysis. In turn and as mentioned above, generative methods (like deep variational autoencoders) can describe variation, with the potential to generate new fruit genotypes in silico, which may be useful for applying new breeding strategies. It is important to note that these methods can also be applied to leaves, flowers, and roots, which may have an even greater diversity of shapes compared to fruits. Therefore, having different complementary analyses available offers an important advantage to better understand the complexity of shape.

Describing internal color patterns is challenging, mainly because color is a quantitative multichannel character. We addressed this problem by defining three reference colors named as “quasired,” “orange-like,” and “pale.” We then automatically determined the percentage of color corresponding to each of these reference colors for each fruit using an autoencoder for fruit denoising and a *k*-means for segmentation. The algorithm calculates the Euclidean distance between the three RGB coordinates obtained by means of clustering to the target color coordinates and classifies the cluster as belonging to one of the three targets whose distance is minimal. The color patterns are satisfactorily dissected, as can be seen in some picked images from the database (Figure 6).

The phenotype results from a complex interaction between the genotype and environmental factors. Portraying the phenotypes would not be worthwhile for breeding if the desirable characters could not be transmitted to the progeny. Thus, quantifying the heritability of all of these traits is crucial. Typically, genetic variance is decomposed in additive and nonadditive effects [76]. Clonally propagated species like strawberry allows direct utilization of dominance and epistatic interaction. We used Bayesian modeling to estimate both additive and dominance effects. As can be observed in Figure 7 and Supp. Table 4, most traits are moderately heritable, and a high degree of variance is explained by the dominance component. In this scenario, prediction accuracy in genomic selection could possibly increase by including dominance in the model [63].

Nevertheless, data are from a single sampling season, making it not possible to estimate the variance caused by genotype × environment (G × E). Therefore, heritabilities reported are likely overestimated. Further, the pedigree utilized considered only parents and offspring, while parents themselves are related, which was ignored except in a subset of parents. The effect in this case should be smaller than that of G × E and should affect the variance of the estimates rather than bias, since relationships decrease quadratically with generation, and most information is contained in closest relatives [77].

We estimated heritabilities using pedigree information, but a similar study could be carried out if genetic markers were available. This would have the extra benefit of allowing to perform genome-wide association studies (GWAS) and to implement genomic selection [63, 76]. It is straightforward to implement these features in our pipeline. Association studies in humans, apple, or tomato have revealed genes or markers associated with human craniofacial shape [34, 78, 79], leaf variation [80], and tomato morphology [29]. To the best of our knowledge, there is not a similar study in strawberry and there is still a long way to go to fully unravel the genetic basis of strawberry shape [81].

## 5. Conclusion

There is a need to develop analysis pipelines for plant high-throughput phenotyping, suitable to automate processes that are often subjective and time-consuming. Our workflow establishes a proof of concept in strawberry morphometrics, which can be transferred to other visual phenotypes and fruits with relatively minor modifications. We developed a python-based pipeline (https://github.com/lauzingaretti/DeepAFS/blob/main/main.ipynb) that shows how to apply our methodology to other fruits like apples, tomatoes, citrus, and prunus. This code is able to automatically read the fruit image, to segment it, and to compute some linear and color descriptors. This code also allows to save the segmented images into a predefined folder, as well as the fruit outline reference points used for posterior multivariate comparison. Overall, our results show that, although fruit shape is made up of a complex set of traits, it can be quickly and automatically evaluated and is moderately heritable (Figures 1, 2, and 7). Future improvements are still needed as, e.g., image segmentation is not always simple in field conditions and many additional phenotypes are of commercial interest (e.g., uniformity, blemishes). Future improvements should also address additional technological developments such as spectral and MIR images [17] and 3D imaging [71]. Finally, a word of caution is that the user should be aware that artificial intelligence tools need thorough training in the specific conditions on which they are going to be employed and that optimizing algorithms may not be that simple.

## Data Availability

Code is available at https://github.com/lauzingaretti/DeepAFS.

## Supplementary Materials

Supplementary Materials Algorithm 1: create a segmented fruit database from raw data. Supp Table 1: scheme of the crosses used in the experiment. Supp Table 2: scheme of the pedigree used in the analysis. Supp Fig. 1: output plot from Procrustes principal component analysis (Proc-PCA). The analysis shows a variation between “elongated” and “globose”-like shape. Supp Table 3: output from Procrustes ANOVA, which evaluates the effect of the crosses on fruit shape. Supp Fig. 2: output from elliptical Fourier analysis. Supp Fig. 3: shape variation derived from PCA on elliptical Fourier analysis. Supp Fig. 4: clustering on the elliptical Fourier components characterizing fruit shapes. Supp Fig. 5: silhouette analysis for number of clusters on shape latent space from variational autoencoder output. Supp Fig. 6: left: silhouette plot for each cluster (0 and 1); right: visualization of the clustered data in the latent space. Supp Fig. 7: the upper panel shows two examples of fruit belonging to the cluster. Suppl. Table 4: heritability values for all the shape- and color-related traits and the additive and dominant components were evaluated.
