# Corrigendum to “Automatic Fruit Morphology Phenome and Genetic Analysis: An Application in the Octoploid Strawberry”

**DOI:** 10.34133/2022/9873618

**Published:** 2022-01-20

**Authors:** Laura M. Zingaretti, Amparo Monfort, Miguel Pérez-Enciso

**Affiliations:** ^1^Centre for Research in Agricultural Genomics (CRAG), CSIC-IRTA-UAB-UB, 08193 Bellaterra, Barcelona, Spain; ^2^Institut de Recerca i Tecnologia Agroalimentàries (IRTA), 08193 Barcelona, Spain; ^3^ICREA, Passeig de Lluís Companys 23, 08010 Barcelona, Spain

In the article titled “Automatic Fruit Morphology Phenome and Genetic Analysis: An Application in the Octoploid Strawberry” [1], some funding information was omitted. The funding DOI “10.13039/501100011033” was missing. The corrected Acknowledgements section is provided below.
